# Vacuum-assisted closure versus on-demand relaparotomy in patients with secondary peritonitis—the VACOR trial: protocol for a randomised controlled trial

**DOI:** 10.1186/s13017-022-00427-x

**Published:** 2022-05-26

**Authors:** Pooya Rajabaleyan, Jens Michelsen, Uffe Tange Holst, Sören Möller, Palle Toft, Jan Luxhøi, Musa Buyukuslu, Aske Mathias Bohm, Lars Borly, Gabriel Sandblom, Martin Kobborg, Kristian Aagaard Poulsen, Uffe Schou Løve, Sophie Ovesen, Christoffer Grant Sølling, Birgitte Mørch Søndergaard, Marianne Lund Lomholt, Dorthe Ritz Møller, Niels Qvist, Mark Bremholm Ellebæk, The VACOR study group, Jens Michelsen, Uffe Tange Holst, Sören Möller, Palle Toft, Jan Luxhøi, Musa Buyukuslu, Aske Mathias Bohm, Lars Borly, Gabriel Sandblom, Martin Kobborg, Kristian Aagaard Poulsen, Uffe Schou Løve, Sophie Ovesen, Christoffer Grant Sølling, Birgitte Mørch Søndergaard, Marianne Lund Lomholt, Dorthe Ritz Møller, Niels Qvist, Mark Bremholm Ellebæk

**Affiliations:** 1 Research Unit for Surgery, Odense University Hospital, Odense, Denmark; 2 Research Unit for Anaesthesiology, Odense University Hospital, Odense, Denmark; 3 OPEN, Open Patient Data Explorative Network, Odense University Hospital and Department of Clinical Research, University of Southern Denmark, Odense, Denmark; 4 Surgical Department, Hospital of Southwest Jutland, Esbjerg, Denmark; 5 Surgical Department, Holbæk Hospital, Holbæk, Denmark; 6 Karolinska Institute, Stockholm, Sweden; 7 Surgical Department, Kolding Hospital, Kolding, Denmark; 8 Surgical Department, Viborg Hospital, Viborg, Denmark; 9 Surgical Department, Aarhus University Hospital, Århus, Denmark; 10 University of Southern Denmark, Odense, Denmark

**Keywords:** Secondary peritonitis, Faecal peritonitis, Vacuum-assisted closure, Primary abdominal closure, Relaparotomy on-demand

## Abstract

**Background:**

Secondary peritonitis is a severe condition with a 20–32% reported mortality. The accepted treatment modalities are vacuum-assisted closure (VAC) or primary closure with relaparotomy on-demand (ROD). However, no randomised controlled trial has been completed to compare the two methods potential benefits and disadvantages.

**Methods:**

This study will be a randomised controlled multicentre trial, including patients aged 18 years or older with purulent or faecal peritonitis confined to at least two of the four abdominal quadrants originating from the small intestine, colon, or rectum. Randomisation will be web-based to either primary closure with ROD or VAC in blocks of 2, 4, and 6. The primary endpoint is peritonitis-related complications within 30 or 90 days and one year after index operation. Secondary outcomes are comprehensive complication index (CCI) and mortality after 30 or 90 days and one year; quality of life assessment by (SF-36) after three and 12 months, the development of incisional hernia after 12 months assessed by clinical examination and CT-scanning and healthcare resource utilisation. With an estimated superiority of 15% in the primary outcome for VAC, 340 patients must be included. Hospitals in Denmark and Europe will be invited to participate.

**Discussion:**

There is no robust evidence for choosing either open abdomen with VAC treatment or primary closure with relaparotomy on-demand in patients with secondary peritonitis. The present study has the potential to answer this important clinical question.

***Trial Registration*:**

The study protocol has been registered at clinicaltrials.gov (NCT03932461). Protocol version 1.0, 9 January 2022.

**Supplementary Information:**

The online version contains supplementary material available at 10.1186/s13017-022-00427-x.

## Background

Perforation of the gastrointestinal tract is the most common cause of secondary peritonitis [1, 2]. The underlying conditions may be appendicitis, anastomotic dehiscence, perforated diverticulitis, intestinal ischemia, or gastroduodenal ulcer, being the most common [3–7]. Faecal peritonitis is associated with high mortality and morbidity rate, with a reported 28-days mortality up to 20%, increasing to 32% at six-month follow-up [8]. Age, comorbidity, time to intervention, and the extent of peritonitis are important risk factors [7, 9–14]. A prerequisite for non-failure is sufficient source control, antibiotics, and in cases with organ dysfunction, postoperative intensive care treatment may be necessary [15–19]. Despite sufficient treatment, the risk of postoperative abdominal complications is high, and several patients may undergo a reoperation (s) to reveal and treat the complications. Another risk is the development of abdominal compartment syndrome. To manage this, three different strategies may be employed: a planned relaparotomy (PR), a relaparotomy on-demand (ROD), or the open abdomen (OA) principle [2, 5, 20–23]. A randomised controlled trial on PR versus ROD in patients with secondary peritonitis due to gastrointestinal perforation, including 232 patients, showed no significant difference in 1-year mortality (36% vs. 29%) and morbidity (44% vs. 40%) [20]. ROD resulted in significantly fewer relaparotomies and lower hospital-related healthcare costs.

A guideline [19] and consensus report [24] from the World Society of Emergency Surgery recommend ROD or VAC as the preferred treatment strategies for intra-abdominal infections with peritonitis and non-traumatic abdominal emergencies. The benefit of primary abdominal closure and ROD strategy is that patients do not require further scheduled operations. The risk is a delay in treatment for ongoing abdominal sepsis, other serious complications, and abdominal compartment syndrome, which may be difficult to recognise clinically in the severely ill patient [25–28]. Delays in treating severe complications might increase the risk of morbidity and mortality [9, 11, 14]. The advantages of VAC are a planned inspection of the abdominal cavity and the possibility to diagnose and treat potential or overt abnormalities on time. The risk is the development of enteroatmospheric fistula, difficulties in abdominal wound closure, and the development of an incisional hernia [29, 30].

The method with VAC was initially introduced in damage control trauma surgery and has gained increasing use in the treatment of complicated intra-abdominal infections [21, 31–43]. A systematic review and meta-analysis by Atema et al. in 2015 showed an incidence of 14.6% for enteroatmospheric fistula (EAF) and 48.5% for incisional hernias in the group treated with VAC, where 82% of the population was treated for peritonitis. The mortality varied between 21.5 and 30.0%. The VAC procedure can be applied and modified in several ways. The most common are fascial traction methods (mesh mediated or non-mesh mediated), the applied vacuum pressure and the interval for changes of the VAC [42]. The VAC treatment in the present study will be performed with a non-mesh-mediated fascial traction method and narrowing technique. A retrospective study from our institute using this technique, including 115 patients with secondary peritonitis, found a mortality rate of 17% and EAF of 3.5%; secondary closure was obtained in 92% of the patients [44].

The present study will aim to compare the postoperative results of ROD and VAC in patients with secondary peritonitis by a randomised controlled trial with peritonitis-related complications as the primary outcome; comprehensive complication index (CCI), mortality, quality of life, the development of incisional hernia, and hospital care utility and costs were the secondary outcomes.

## Objectives

- Primary
  - Peritonitis-related complications (Table 1) within 30 or 90 days and one year after index surgery
- Secondary
  - CCI within 30 or 90 days and one year after index surgery
  - Mortality within 30 or 90 days and after one year
  - SOFA score and C-reactive protein (CRP) measured in the first seven days after index laparotomy
  - Incisional hernia rate after 12 months assessed by clinical examination and abdominal CT-scan
  - Quality of life after 3 and 12 months assessed by the SF-36 questionnaire
  - Hospital care utility within three months after index surgery (Table 2)
- Tertiary
  - The concentration of lactate, glycerol, pyruvate, glucose, and cytokines in the peritoneal fluid in a subgroup of 10 patients from each group measured by intraperitoneal microdialysis on postoperative days 0–4

**Table 1:** Peritonitis-related complications

Disease-related major morbidity needing readmission and conservative treatment but not surgery
Fistula: non-anatomical connection between intestine and cutis, communication between GI tract and external atmosphere or between 2 hollow organs
Wound dehiscence/incisional hernia with obstruction: full-thickness discontinuity in the abdominal wall with bulging of abdominal content
Abscess needing percutaneous drainage: pus-containing non-pre-existing cavity confirmed by positive Gram stain or culture
Renal failure: urine production < 500 mL/24 h with rising levels of blood urea nitrogen and creatinine combined with dehydration (decreased circulating volume with elevated haematocrit needing intravenous rehydration) based on inadequate oral intake, nausea/vomiting, or both (only when needing readmission)
Myocardial infarction (electrocardiogram and enzyme changes suggestive of myocardial infarction or needing admission to coronary care unit), pulmonary embolus (ventilation-perfusion mismatch on lung scintigraphy), or cerebrovascular accident (ischemic or non-ischemic with persistent paresis or paralysis without previous history)
Gastric or duodenal bleeding: needing endoscopic treatment or embolisation therapy
Respiratory failure due to pneumonia, pleural effusion, or pulmonary oedema and needing oxygen therapy or mechanical ventilation
Urosepsis: urinary tract infection with positive urine and blood cultures and circulatory shock

Disease-related major morbidity needing surgical intervention during first admission or readmission
Incisional hernia: full-thickness discontinuity in abdominal wall with bulging of abdominal contents with or without obstruction with disabling concerns interfering with daily activities
Bowel obstruction or herniation due to intra-abdominal adhesions: diagnosis must be confirmed during surgery
Burst abdomen: complete midline or transverse discontinuity in abdominal wall
Abdominal compartment syndrome: intra-abdominal hypertension ≥ 25 mm Hg with tense abdomen and with increasing respiratory failure, renal failure, or both, measured by the urinary bladder pressure method (modified Burch criteria)
Fistula: non-anatomical connection between intestine and cutis, communication between GI tract and external atmosphere or between 2 hollow organs
Intra-abdominal bleeding: only when septic bleeding after index laparotomy or relaparotomy or surgical bleeding after relaparotomy but not after index laparotomy
Intra-abdominal haematoma needing surgical evacuation
Perforation of visceral organ confirmed at surgery
Anastomotic leakage: anastomotic leak on contrast imaging needing surgery or contrast-enhanced computed tomography scan, confirmed at relaparotomy
Ischemia or necrosis of a visceral organ: critically reduced blood flow to an intra-abdominal organ causing tissue loss, confirmed at pathological examination
Enterostomy dysfunction due to prolapse, stenosis, or retraction
Gastric or duodenal ulcer bleeding needing intervention of any type

Reference Table 1: van Ruler O, Mahler CW, Boer KR, Reuland EA, Gooszen HG, Opmeer BC, de Graaf PW, Lamme B, Gerhards MF, Steller EP, van Till JW, de Borgie CJ, Gouma DJ, Reitsma JB, Boermeester MA; Dutch Peritonitis Study Group. Comparison of on-demand vs planned relaparotomy strategy in patients with severe peritonitis: A randomised trial. JAMA. 2007 Aug 22;298(8):865–72. Available from: http://dx.doi.org/10.1001/jama.298.8.865

**Table 2:** Health care utility

Length of admission at ICU (Total number of days in a three-month period)
Length of admission at ward (Total number of days in a three-month period)
VAC—Time from index operation to primary closure
Total amount of VAC dressing changes (number of times)
Number of scheduled VAC changes
Number of re-operations with VAC
Number of re-operations
Number of radiologic interventions during admission(s)
Number of computed tomography scans after index operation
Number of days alive outside the ICU in a three-month period

Although the validated CCI may be the best parameter to monitor postoperative complications and morbidity, there are no reliable data in the literature on CCI from this specific group of patients. Therefore, we could not make a meaningful sample size calculation based on CCI alone. Peritonitis-related complications described in the RCT by Van Ruler et al. were the best estimate for the postoperative complications and morbidity we could find in the literature and were used for the study's sample size calculation [20].

## Methods

### Study design

A multicentre non-blinded superiority randomised controlled trial on VAC vs. ROD. Danish, as well as other European centres, will be invited to participate. The study protocol adheres to the guidelines determined in Standard Protocol Items: Recommendations for Interventional Trials (SPIRIT) (Additional file 2) [45].

The VACOR study will include two separate studies: VACOR-Main and VACOR-Microdialysis.

All centres must participate in the VACOR-Main study, with only Odense University Hospital participating in the VACOR-Microdialysis study. VACOR-Main will report on all primary and secondary outcomes; in VACOR-Microdialysis, a sub-study of 10 patients from each group will be included. A microdialysis catheter will be placed in the peritoneal cavity, and samples of the peritoneal fluid will be collected every 6th hour to measure the concentration of lactate, glucose, pyruvate, glycerol, cytokines (IL-1b, IL-6, IL-10, TNF-a) and metalloproteins (MMP9 and MMP8). The purpose is to investigate intraperitoneal metabolic changes and inflammatory responses in the two groups.

### In-hospital healthcare utility

Three months after the index operation, a record review will be made to estimate the healthcare utility. All data will be retrieved from the electronic patient system and include surgeries, total hospital stay, admissions to the ICU, and radiological interventions (Table 2). For the analysis, unit costs were obtained from the Danish Health Authority. The diagnosis-related groups (DRG) will be used, which express the hospital's average operating expenses within each DRG group. To ensure comparability between centres, we will utilise the Danish costs for both Danish and international patients.

### Study population

#### Inclusion criteria

Patients eligible for enrolment are 18 + years of age and scheduled for acute laparotomy due to suspected peritonitis originating from perforation of the small bowel, colon, or rectum. To be included, purulent, enteric, or faecal contamination in a minimum of 2 out of 4 quadrants must be confirmed.

#### Exclusion criteria

- Diffuse peritonitis originating from a perforation on the stomach, duodenum, gallbladder, appendix, necrotising pancreatitis, salpingitis, or peritoneal dialysis
- Primary peritonitis
- Immunocompromised (history of steroid or biological treatment within the last three months or previous organ transplantation)
- Chronic parenchymal liver disease (chronic liver disease with plasma bilirubin above 35 mmol/L)
- Pregnancy
- Patients with end-stage disease (metastatic disease)
- Laparoscopic surgery (not converted to laparotomy)
- Acute occlusion of superior mesenteric artery
- Peritoneal carcinomatosis
- Abdominal trauma
- Lack of consent from the surgical equipoise
- Local peritonitis confined to one quadrant only

### Study setting

The study will be conducted in general surgery departments, emergency departments, and operating theatres. The lead centre is Odense University Hospital in Odense, Denmark, a tertiary referral academic medical centre. Other recruiting sites will include academic and community hospitals located in Europe, familiar with the interventions and willing to adhere to the treatment regimens.

### Randomisation

**Fig. 1:**
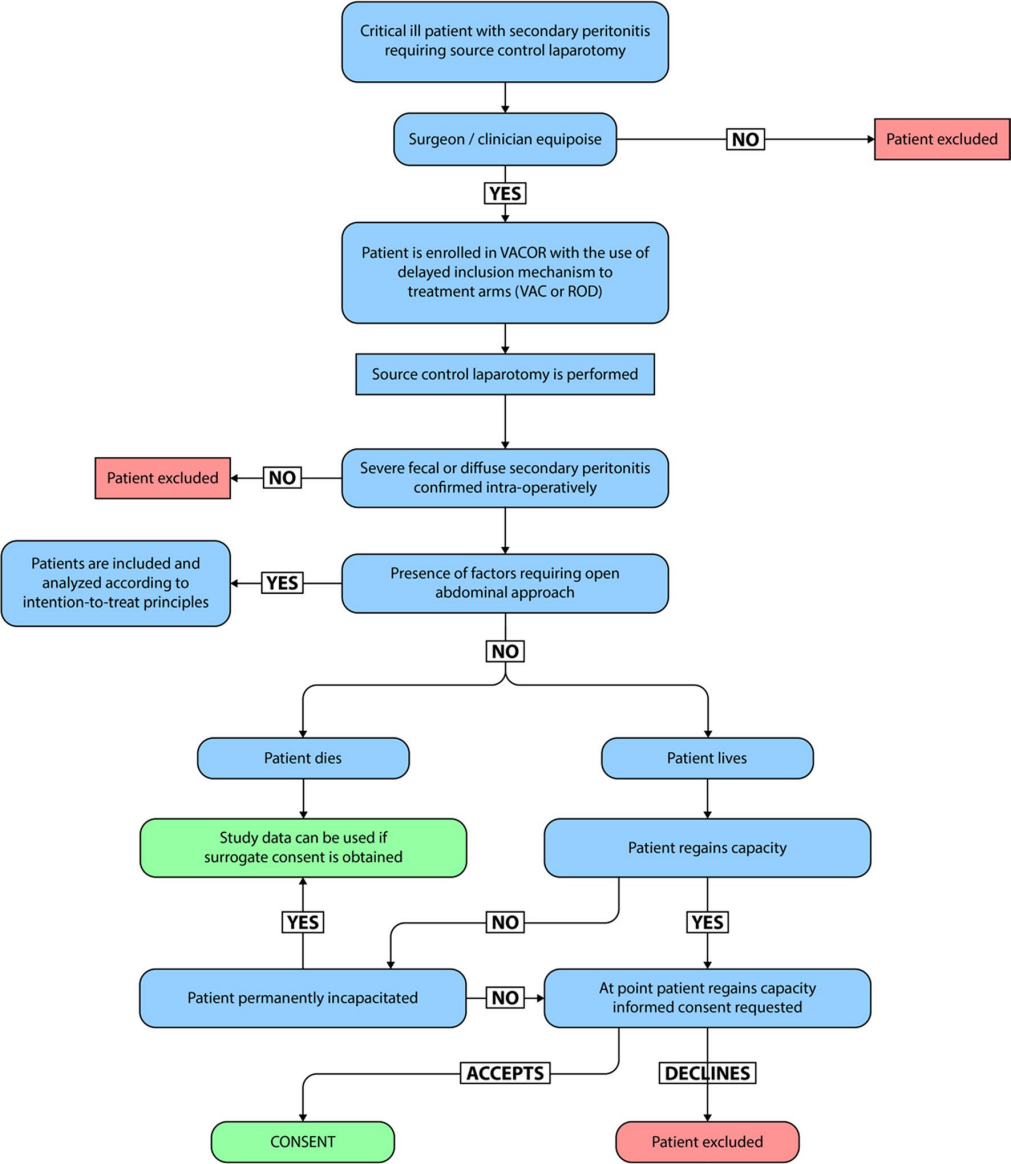
Flowchart of inclusion and obtaining consent by the surgical equipoise

**Fig. 2:**
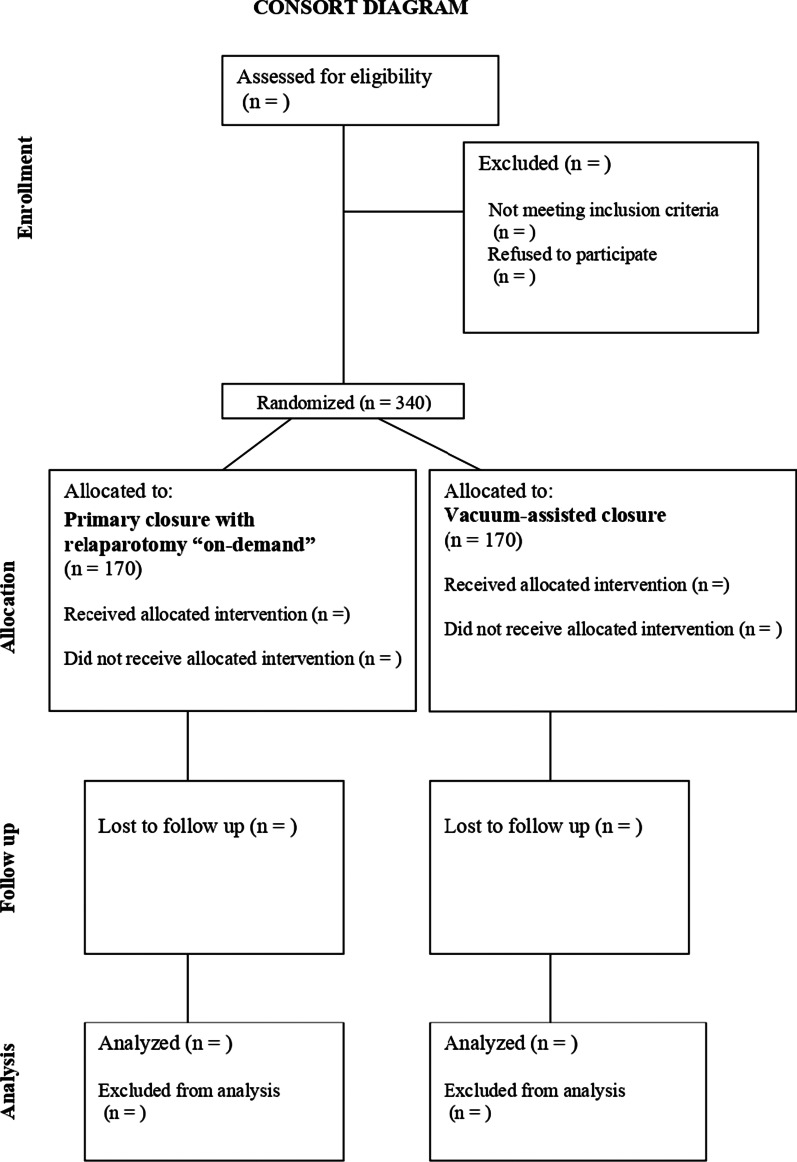
CONSORT flow diagram

Patients are included by a surgical equipoise followed by patient information and consent after recovery. The on-call surgeon will contact the primary investigator from each site when a patient is scheduled for diagnostic laparoscopy or explorative laparotomy on suspicion of secondary peritonitis. Patients fulfilling the inclusion criteria will be randomised after consent has been obtained by the surgical equipoise (Fig. 1). This could occur before, during, or at the end of index laparotomy, disclosing a complete overview of all potential candidates. Most patients will be able to receive both treatments regardless of what arm they are allocated without changing the treatment regimen. However, there will be some exceptions where it is necessary to modify or change the surgical treatment, irrespective of the randomisation allocation. Centres that do not receive approval from their respective scientific ethics committee to include patients through a surgical equipoise must obtain informed and written consent before randomisation. Randomisation will be web-based via (REDCap ®) in blocks of 2, 4, and 6 stratified for centre and age above or below 65 years. The justifications for stratifying according to the centre are that there might be differences in the surgical treatment, preoperative optimisation, and postoperative treatment, both in the ward and intensive care unit (ICU), which might affect the outcome. The patients will be randomised to either abdominal closure with ROD or open abdomen with VAC according to transparent reporting of trials (CONSORT) (Fig. 2). The randomisation tool and eligibility criteria can be accessed through our website, www.vacor.sdu.dk.

In cases where the surgeon finds that the allocated treatment may be contraindicated or is judged to harm the patient, it will be left to the surgeon's discretion to choose the most appropriate treatment. In cases where the bowels are left stapled in discontinuity as a part of the damage control principle, the patient's fascia cannot be closed due to swelling or the presence of abdominal compartment VAC treatment may be applied irrespective of the randomisation. These patients remain in the trial for an intention-to-treat and per-protocol analysis. By excluding such patients, there might be a risk of selection bias. Any eligible patient not included will be registered in a screening log.

### Preoperative patient assessment

The patients are preoperatively assessed according to Sequential Organ Failure Assessment (SOFA) to determine the degree of organ dysfunction [46].

### Interventions

#### Vacuum-assisted closure

The VAC® Abdominal Dressing System (KCI Vacuum Assisted Closure, San Antonio, TX, USA) will be used [44]. A video illustrating the procedure is attached in the supplementary material (Additional File 1). Intestines, including lateral aspects, are covered by the visceral protective layer. The first layer of foam is placed in the laparostoma on top of the visceral protective layer and must extend below the fascia at a distance of 5 cm from the fascial opening. Above this, a minimum of one piece of foam is folded and placed in the laparostoma. Finally, the laparostoma will be covered by the occlusive drape. A circular opening of approximately 5 cm in diameter will be created in the drape where the connection tubes to the vacuum pump will be placed. Simultaneously, while applying the negative pressure of 125 mmHg, the wound edges are approximated manually towards the midline. The dressing will be changed at an interval of approximately 48 h as standard or whenever needed according to the clinical condition. Each dressing change must be performed with the patient under general anaesthesia and muscle relaxation in the operating theatre. Peritoneal fluid must be cultured at each dressing change and when the fascia is closed. The fascial closure can, in some instances, be difficult due to swelling or the physical inability to close the abdomen. It must commence as soon as possible, judging by intra-abdominal findings, gastrointestinal function, and renal function. The aim will be to close the abdomen within eight days after the index operation.

The fascia closure after VAC treatment can be according to Israelsson's principle, as described below, or at the surgeon's discretion. A staged closure may start distally, proximally, or in combination. To ensure the uniformity of intervention in the arm receiving the open management, we have produced a video where the application of the VAC system is demonstrated in a step-by-step manner, as well as providing training for centres not familiar with the technique.

#### Primary closure

The Israelsson principle includes a running suture of the fascia with a distance of 5 mm between the stitches of 5 mm and the distance to the fascial edge of 5–10 mm. Monofilament PDS 2–0 or equivalent is used [47]. The suturing is started cranially and caudally, and the sutures are tied with self-locking knots. Approximately four times as much suture material as the length of the wound must be used or more. The peritoneal fluid must be cultured at closure.

#### Relaparotomy on-demand (ROD)

The treating surgeon decides at daily rounds whether a ROD is required and should be guided by the patient's general condition, gastrointestinal function, renal function, imaging findings, drain findings, and inflammatory parameters. A planned relaparotomy will not be considered a complication.

#### Microdialysis

The intraperitoneal microdialysis catheter will be placed before the abdominal closure or before applying the VAC system. The microdialysis catheter (M-dialysis 63, Microdialysis AB, Stockholm, Sweden) will be introduced through the abdominal wall outside the laparostoma via a charrier ten split cannula and placed in the peritoneal cavity between small intestine loops. The catheter will be perfused by an isotonic perfusion fluid (Perfusion fluid CNS Dextran, Microdialysis AB, Stockholm, Sweden) via a small pump (Microdialysis CMA 106 pump, Microdialysis AB, Stockholm, Sweden) at a flow rate of 0.3 μl/min. The catheter will be anchored to the skin. Samples will be collected in vials at 6-h intervals for the first four postoperative days. Bedside analysis for lactate, glycerol, and pyruvate concentration will be made via the ISCUSflex Microdialysis Analyzer (Microdialysis AB, Stockholm, Sweden). After analysis, the samples will be stored at  − 80 °C to analyse cytokine and MMP concentrations.

### Postoperative patient assessment

Immediately after index operation, the surgeon fills out the baseline form containing patient characteristics, Charlson comorbidity index, aetiology and extent of the peritonitis, surgical procedure, Mannheims peritonitis index and classification of the OA according to Bjork's classification [48, 49]. Patients can be transferred postoperatively to the intensive care unit (ICU) or the ward at the discretion of the treating team. Upon arrival to either the ward or the ICU, Acute Physiology and Chronic Health Evaluation II (APACHE II) score must be obtained by the attending anaesthesiologist [46, 50]. SOFA-scoring and routine blood samples with CRP, bilirubin, creatinine, and platelets must be performed daily within the first seven days after index operation. Discharge from the ICU will be at the discretion of the attending intensivist and surgeon.

### Follow-up

At hospital discharge, the patients will be booked for follow-up after 12 months in the outpatient clinic for abdominal palpation and abdominal CT-scan with intravenous contrast and Valsalva manoeuvre. In addition, the SF-36 questionnaire will be completed at the 3- and 12-month follow-up.

#### Data collection and participant timeline

**Fig. 3:**
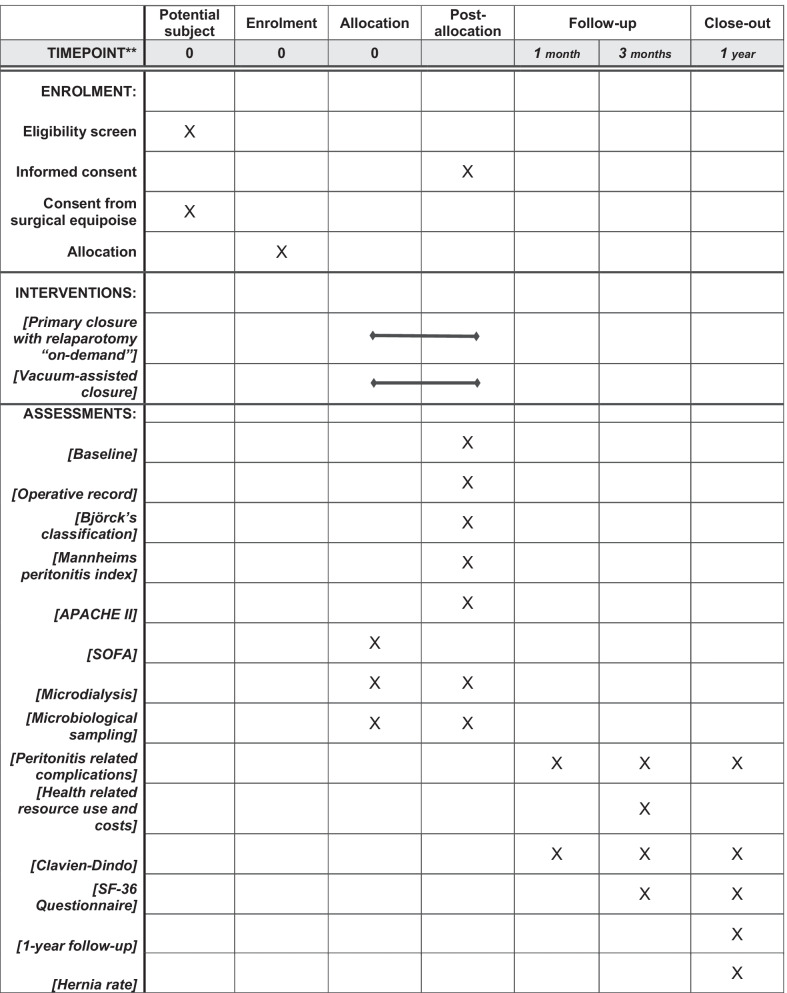
Participant timeline

The pre- and perioperative assessment will consist of: baseline data (sex, age, surgery date, height, weight, body mass index, ASA score, WHO performance score, smoking, alcohol consumption, presence of comorbidities, previous abdominal surgeries, and steroid use). Data for surgical findings include aetiology of disease, anatomical location of intestinal perforation, degree of contamination, surgical treatment, method of abdominal wall closure, and suture material used for abdominal wall closure. Postoperative monitoring consists of SOFA, APACHE II, CRP, VAC treatment duration, unplanned VAC change, and the number of laparotomies in the ROD group. A patient record review will be performed at the follow-up after one month, three months, one year, and five years (Fig. 3). All data will be stored in REDCap®, hosted by the Odense Patient data Explorative Network (OPEN).

#### Sample size and power

With an expected peritonitis-related complications rate of 40% in the ROD group [20] and 25% in the VAC group [42, 44], the desired power of 80%, a significance level of 0.05, and an expected drop-out of 5%, a total of 340 patients should be included.

As the CCI distribution in this group of patients is unknown, we could not perform an explicit sample size calculation for this secondary outcome. A 0.32 standard deviation difference in mean CCI between the two groups could be detected with 80% power with this sample size.

To ensure sufficient recruitment, the study will be multicentre and European. Eight active centres have been included, and two are in process. Randomisation tools along with eligibility criteria are accessible through our website. The workflow and relevant contact details appear on posters at the participating departments. Study progress will be available on the website.

#### Statistical analysis

Patient characteristics will be summarised with frequencies and proportions (for categorical variables) or with mean values ± standard deviation, median values, quartiles, and minimum and maximum values (for numerical variables). Categorical variables will be compared using a Fisher's exact test and continuous variables with a Wilcoxon rank-sum test.

The primary peritonitis-related complication outcome will be compared between intervention groups by the Chi-square test, reported as relative risk with a 95% CI. The CCI outcome will be compared by linear regression with bootstrapped standard errors reporting the mean difference with 95% confidence intervals.

The main analysis will be performed as a superiority analysis of VAC treatment against primary closure with ROD. In addition, a non-inferiority analysis with a margin of 5% will be reported for peritonitis-related complications as a secondary analysis.

A univariate analysis will be performed on the individual complication types (abscess, leakage, etc.) and complications as a whole (peritonitis-related complications and CCI). Fisher's exact or Chi-square test will be used to compare the treatments depending on the number of observations.

Adjusted analysis by logistic regression will be performed for complications as a whole and the individual complications as an outcome, adjusted for age, performance status, and comorbidity. The above analyses will also be performed as a subgroup analysis where patients with APACHE II score > 10 will be included. This evaluates VAC and ROD in the most seriously ill portion of the patient population.

The hospital healthcare utility and average treatment costs are compared between the treatment groups. The resource use will be reported as the mean difference with 95% confidence intervals (CI) compared by linear regression. In case of deviations from normality assumptions, bootstrapping with 1000 repetitions will be performed. Finally, the proportion of patients who experience radiological, acute operations will be compared by binomial regression estimating relative risk (RR) with 95% CI.

The interim analysis will be performed at 25%, 50%, and 75% of recruited patients on the primary outcome after 30-days to detect significant differences between groups at the earliest possible time, ultimately leading to the termination of the study. We have adjusted our power calculation to the interim analyses using the O'Brien–Fleming method. The study group will have access to the results of the interim analyses and may make the final decision to terminate the study.

All of the above analyses will be performed as both intention-to-treat (patients will be analysed according to their randomisation group) and per-protocol analysis (what actually happened). The main analyses will be performed as complete case analyses. Multiple imputations will impute missing values in a supplementary analysis, including baseline characteristics as predictors.

*P* values < 0.05 will be considered statistically significant. Statistical calculations will be performed using Stata software (version 15, Stata Corp LP, Texas, USA).

In the VACOR-Microdialysis sub-study, the parameters will be compared using descriptive statistics for continuous and discrete variables. Repeated measurement across time points will be compared by mixed-effects regression models, including the interaction between time points and operation method and a random intercept for each patient. In addition, normality assumptions will be graphically assessed using quantile–quantile plots.

#### Dissemination policy

The study results will be published in scientific international peer-review journals and presented at relevant conferences. Results will be available to participants, healthcare professionals, the public, and other relevant groups in an anonymous patient format. The study protocol will be publicly accessible. Authorship eligibility adheres to Vancouver conventions guidelines.

## Discussion

The VACOR trial addresses several important unanswered clinical questions in the surgical treatment of complicated intra-abdominal infections with special reference to the choice of primary abdominal closure with ROD or the open abdomen with VAC treatment.

In some conditions such as severely complicated peritonitis, second look for ischemia and septic shock, where patients have substantial visceral oedema with high risks of abdominal compartment syndrome, establishing an OA with VAC may be preferred [5, 17, 19, 24]. According to intention-to-treat and per-protocol principles, these patients will be included in the present study. The OA with VAC treatment may have several benefits, including drainage of residual infection, preventing intra-abdominal compartment syndrome, and the timely treatment of complications. The disadvantages are incisional hernia and EAF [23, 42, 44, 51–55]. A recent review of temporary abdominal closure techniques found a higher incidence of EAF in septic than non-septic patients (12.1% vs. 3.7%, respectively) [56]. A large cohort study by Coccolini et al. [57], including 649 patients treated with OA where most patients had peritonitis, could not confirm that peritonitis or temporary abdominal closure with or without negative pressure was related to the incidence of EAF. In systematic reviews with meta-analyses and non-randomised studies it has been shown that VAC is the safest of all temporary abdominal closure techniques [21, 31–43].

In a study including patients with severe secondary peritonitis, patients were randomised to OA with non-resorbable polypropylene mesh versus primary closure with ROD [58]. The study was discontinued at the first interim analysis. The study showed an insignificant higher mortality risk in the OA group of 55% compared to 30% in the ROD group. However, there was a relative risk of 1.83 and an odds ratio of 2.85 in the OA group. Hence, the authors concluded a tendency towards a better outcome in the ROD group.

Animal and in silico studies suggest that VAC treatment suppresses systemic inflammatory reactions and prevents multi-organ failure by draining the peritoneal fluid [59, 60]. In a small animal study comparing VAC to passive drainage after inducing abdominal sepsis, the mortality rate was 17% versus 50%. This difference was not statistically significant (*p* = 0.19), likely due to the small number of included animals [60]. In the only human RCT, levels of plasma and peritoneal cytokines in patients with abdominal trauma and intra-abdominal sepsis were compared [61]. Participants were allocated to Barker's or vacuum pack. The study revealed no significant difference in bio-mediator levels or peritoneal drainage. However, a significant difference was observed in 90-day mortality (21.7% vs. 50%), favouring the VAC group. No human RCT studies compare bio-mediators in VAC versus primary closure with ROD.

Diagnosing postoperative or ongoing abdominal complications with the ROD strategy can sometimes be challenging. The literature suggests that progressive or persistent organ failure in the early postoperative phase is the best indicator of positive findings and ongoing infection [20, 26, 62]. The choice of whether or not to perform a relaparotomy is often subjective, based on local guidelines and personal experiences [25–28]. In a randomised study 112 patients with secondary peritonitis were included in the ROD arm and 113 in the planned relaparotomy arm [20]. A total of 42% of patients in the ROD group received relaparotomies, 71% had negative findings, and 29% had positive findings at the relaparotomy, respectively.

As well as our study, another randomised multicentre trial, the COOL study [63], is actively recruiting patients with secondary peritonitis to either VAC or primary closure. The primary endpoint is the 90-day mortality rate. The COOL study includes severely ill patients, assessed by physiological scores and secondary peritonitis originating from the lower- and upper gastrointestinal tract, gallbladder, and adnexa. The current study includes patients with secondary peritonitis from the lower gastrointestinal tract with purulent, enteric, or faecal contamination in a minimum of two out of four abdominal quadrants, irrespective of the patient's general condition. Therefore, there is a risk that some patients may be overtreated. However, the risk might be low since we have excluded patients with upper GI perforations, appendicitis, pancreatitis, and local peritonitis. The majority of the patients are expected to be septic, and the literature has indicated that these patients may benefit from VAC treatment [17, 19, 24]. There might be a grey zone of precisely which patients may benefit from VAC treatment. The COOL trial includes only cases where the surgeon a priori finds that VAC might be beneficial before the randomisation takes place. Our study explores the generalisability of VAC or abdominal closure as the main principle in patients with diffuse peritonitis from the lower gastrointestinal tract. Our study and the COOL study utilise inclusion via a surgical equipoise, ensuring that a high fraction of eligible patients will be included.

A limitation of the current study is that the power calculation is based on peritonitis-related complications and not the CCI-index due to the literature lacking explicit results for VAC and ROD. The abdominal condition at index operation will be classified according to Bjorck's amended classification [48]. The system was intended for established open abdomens rather than the abdominal condition at the index operation. Nonetheless, it is a novel usage for this reviewer. However, this might be another valuable aspect of our study to comment upon whether the classification is further helpful for general use in acute general surgery.

## Conclusions

There is no robust evidence for choosing either open abdomen with VAC treatment or primary closure with relaparotomy on-demand in patients with secondary peritonitis. The present study has the potential to answer this important clinical question.

## Supplementary Information

**Additional file 1.** Video illustrating the application of the VAC system.

**Additional file 2.** SPIRIT Checklist.

## Data Availability

On request, data can be shared in an anonymised form if a data processor agreement is obtained.

## Abbreviations

VAC: Vacuum-assisted closure
PR: Planned relaparotomy
ROD: Relaparotomy “on-demand”
CCI: Comprehensive complication index
SF-36: Short form survey 36
OA: Open abdomen
EAF: Enteroatmospheric fistula
CT: Computed tomography
CRP: C-reactive protein
CI: Confidence interval
OPEN: Odense Patient data Explorative Network
ASA: American Society of Anesthesiology
APACHE II: Acute Physiology and Chronic Health Evaluation II
SOFA: Sequential Organ Failure Assessment
ICU: Intensive care unit
DRG: Diagnosis-related groups
